# Diabetes and Its Impact on Cardiogenic Shock Outcomes in Acute Myocardial Infarction with Polyvascular Disease: A Comparative Analysis

**DOI:** 10.3390/biomedicines12081900

**Published:** 2024-08-20

**Authors:** Marlon V. Gatuz, Rami Abu-Fanne, Dmitry Abramov, Mamas A. Mamas, Ariel Roguin, Ofer Kobo

**Affiliations:** 1 Department of Cardiology, Hillel Yaffe Medical Center, Hadera 3200003, Israel; mvg.cardio@gmail.com (M.V.G.); rabufanne@gmail.com (R.A.-F.); aroguin@technion.ac.il (A.R.); 2 Department of Cardiology, Linda Loma University Health, Loma Linda, CA 92354, USA; dabramov1@gmail.com; 3 Keele Cardiovascular Research Group, Keele University, Keele ST5 5BG, UK; mamasmamas1@yahoo.co.uk; 4 National Institute for Health and Care Research (NIHR), Birmingham Biomedical Research Centre, Birmingham B15 2TT, UK

**Keywords:** diabetes mellitus, cardiogenic shock, acute myocardial infarction, poly-vascular disease, outcomes

## Abstract

Background: Diabetes mellitus (DM) significantly impacts cardiovascular outcomes, particularly in patients with acute myocardial infarction (AMI) complicated by cardiogenic shock (CS). The presence of polyvascular disease further complicates the prognosis due to the increased burden of atherosclerosis and comorbidities. This study was designed to investigate the combined impact of DM and polyvascular disease on outcomes in patients with AMI and CS. Method: Using the National Inpatient Sample database, we analyzed 39,140 patients with AMI complicated by CS and known polyvascular disease. The patients were stratified by diabetes status. The study assessed in-hospital major adverse cardiovascular and cerebrovascular events (MACCE), mortality, cerebrovascular accident (CVA) and major bleeding. Multivariable logistic regression models were used to examine the association between in-hospital outcomes and diabetes, adjusting for baseline differences. Results: Of the study population, 54% had DM. The patients with DM were younger (69.5 vs. 72.1 years, *p* < 0.001) and more likely to be female (36.7% vs. 34.2%, *p* < 0.001). After adjustment, the patients with DM showed a 17% increased mortality risk (aOR 1.17, 95% CI: 1.11–1.23, *p* < 0.001) and a higher risk of major adverse cardiovascular and cerebrovascular events (aOR 1.05, 95% CI: 1.01–1.10, *p* = 0.020). Conclusions: DM significantly impacts outcomes in patients with AMI complicated by CS and polyvascular disease, leading to increased mortality risk, longer hospital stays, and higher healthcare costs. These findings underscore the need for targeted interventions and specialized care strategies for this high-risk population.

## 1. Introduction

Diabetes mellitus is associated with an increased risk of acute myocardial infarction, with high morbidity and mortality rates [1,2]. Polyvascular disease, defined as the presence of atherosclerosis in two or more major arterial beds, is an independent predictor of major adverse cardiovascular events (MACE) and all-cause mortality in patients with AMI and in those with DM [3,4,5,6].

Cardiogenic shock, a severe condition often complicating AMI, is associated with high in-hospital mortality rates. It is the leading cause of death in patients with AMI, with a 30-day mortality rate of approximately 40% and a 1-year mortality rate of 50% [7]. The interplay between diabetes and CS further complicates the prognosis, as diabetes is known to double the incidence of CS in AMI patients [8,9].

The negative impact of polyvascular disease on the prognosis of patients with DM is well-established [3,4,5]. While there is substantial evidence on the effects of DM on management and outcomes in patients with AMI and CS, important knowledge gaps remain. Specifically, the role of DM in populations with a higher comorbidity burden, particularly those with polyvascular disease, has not been well characterized in the context of AMI complicated by CS. This represents a critical area of investigation that has been largely unexplored.

Therefore, the current study aims to investigate the combined impact and outcomes of DM and polyvascular disease in patients with AMI complicated by CS, which may allow improvements in management strategies and patient outcomes in this high-risk population.

## 2. Methods

### 2.1. Data Source

The National Inpatient Sample (NIS), which has been available since 1988, is one of the largest publicly available all-payer inpatient healthcare databases in the United States. It contains data from around 7 million hospital stays each year, which approximates a 20-percent stratified sample of all discharges from U.S. community hospitals, excluding rehabilitation and long-term acute care hospitals. The NIS is part of the Healthcare Cost and Utilization Project (HCUP), and it intends to produce U.S. regional and national estimates of inpatient utilization, access, cost, quality, and outcomes [10].

### 2.2. Study Design and Population

In this retrospective study, we conducted a comprehensive analysis of adult patients (aged ≥18 years) hospitalized with a diagnosis of AMI complicated by CS, who also had pre-existing DM and polyvascular disease. We utilized the International Classification of Diseases, Tenth Revision, Clinical Modification (ICD-10-CM) codes for patient identification and data collection.

**Figure 1:**
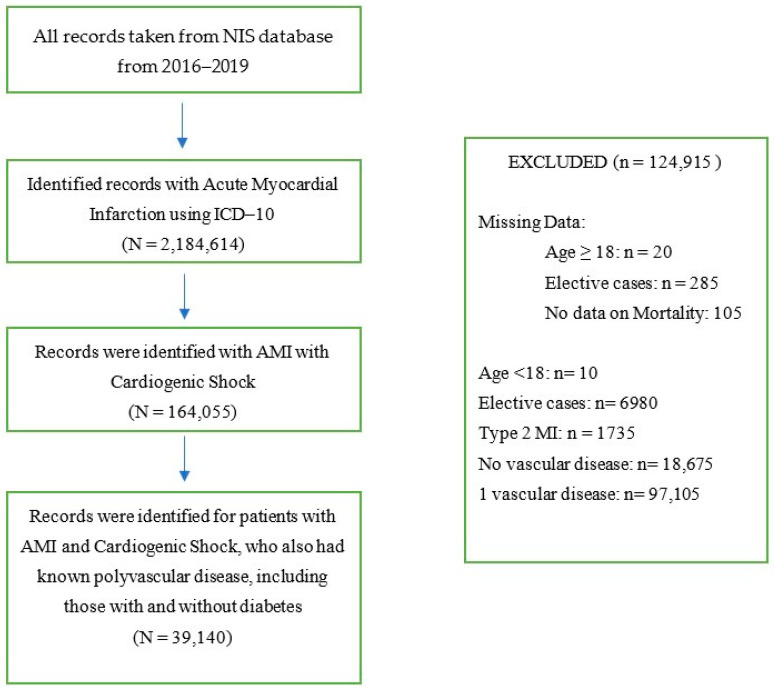
Flow Diagram.

Missing data on age, gender, elective, admission type and day, and mortality status were excluded from the analysis. Patients with type 2 MI, elective admissions, absence of vascular disease, and one vascular disease were also excluded from analysis (see Figure 1 for study flow diagram).

Vascular diseases were defined based on the presence of ischemic heart disease, cerebrovascular disease, renal disease renal disease (excluding nephrotic syndrome and chronic renal calculus), aortic disease, and peripheral vascular disease of extremities [6]. In this study, a “vascular bed” is defined based on the presence of vascular disease, with one vascular bed corresponding to one type of vascular disease, two vascular beds to two types, and so on. Diabetes mellitus includes both Type 1 and Type 2 DM.

The analysis incorporated up to 30 diagnoses per discharge record. We used ICD-10-CM codes to identify vascular diseases, complications, and procedural information during hospitalization. This included data on coronary angiography, percutaneous coronary intervention (PCI), coronary artery bypass graft (CABG), thrombolysis, use of mechanical ventilation, and circulatory support.

Table S1 provides the full list of ICD-10 codes for the patient and procedural characteristics. Patient demographics were recorded for each hospital discharge including age, gender, race, admission day (weekday or weekend), expected primary payer, and median household income according to ZIP code.

### 2.3. Outcomes

The main outcomes evaluated in this study encompassed in-hospital adverse events, including major adverse cardiovascular and cerebrovascular events (MACCE), all-cause mortality, acute ischemic cerebrovascular accident (CVA), and major bleeding. MACCE was defined as a composite of all-cause mortality, acute ischemic CVA or transient ischemic attack, and specific cardiac complications. These cardiac complications included, but were not limited to, coronary artery dissection, pericardial effusion (including tamponade), Dressler’s syndrome, post-MI angina, intracardiac thrombus, and acute mechanical complications. Major bleeding was classified as any occurrence of gastrointestinal, retroperitoneal, intracranial, or intracerebral hemorrhage, as well as periprocedural or unspecified hemorrhage, or the need for blood transfusion. Additionally, we analyzed the utilization rates of invasive management strategies, specifically coronary angiography, PCI, CABG, thrombolysis, use of mechanical ventilation, and circulatory support, to provide a comprehensive overview of the treatment approaches employed.

### 2.4. Statistical Analysis

Statistical analysis was performed on IBM SPSS version 25. Continuous variables were presented as mean, median, and interquartile range, due to skewed data, and categorical data were presented as frequencies and percentages. Categorical variables were compared using Pearson’s chi-squared test, while continuous variables were compared using the Mann–Whitney U test, as appropriate. To ensure accurate representation of the population, we employed sampling weights, as specified by AHRQ, to calculate estimated total discharges. The association between in-hospital outcomes and the number and location of diseased vascular beds was examined using multivariable logistic regression models. Results were expressed as odds ratios (OR) with corresponding 95% confidence intervals (CI). All models were adjusted for baseline differences between the groups, controlling for the following covariates: age, gender, weekend admission, hospital bed size, region and location/teaching status, ST-elevation myocardial infarction (STEMI), CABG, PCI, CA, thrombolysis, mechanical ventilation, circulatory support, ventricular fibrillation (VF), ventricular tachycardia (VT), atrial fibrillation, heart failure (HF), hypertension, valvular heart disease, smoking status, chronic liver disease, anemia, thrombocytopenia, coagulopathies, and malignancies. This extensive adjustment was intended to minimize confounding and provide a more accurate assessment of the independent associations between vascular disease burden and outcomes.

## 3. Results

In the study, the records of 39,140 patients with AMI and CS who also had polyvascular disease were identified. Among these patients, 21,140 (54%) had DM. The baseline characteristics of the patients, stratified by diabetes status, are shown in Table 1. The patients with a diagnosis of diabetes were younger (69.5 vs. 72.1 years, *p* < 0.001) and were more likely to be female (36.7% vs. 34.2%, *p* < 0.001). Those with diabetes had a higher burden of comorbidities, including HF (75.3% vs. 67.8%, *p* < 0.001), hypertension (92.8% vs. 82.3%, *p* < 0.001), chronic kidney disease (78.2% vs. 57.8%, *p* < 0.001), obesity (22.1% vs. 10.8%, *p* < 0.001), chronic liver disease (1.2% vs. 0.9%, *p* < 0.001), and anemia (55.6% vs. 49.2%, *p* < 0.001). However, those with diabetes had lower rates of smoking (37.5% vs. 49.0%, *p* < 0.001), valvular heart disease (23.1% vs. 25.4%, *p* < 0.001), atrial fibrillation (34.7% vs. 37.5%, *p* < 0.001), coagulopathy (5.9% vs. 6.9%, *p* < 0.001), and malignancies (*p* < 0.001). The patients with DM were less likely to be admitted with STEMI (38.2% vs. 50.0%, *p* < 0.001).

### 3.1. In-Hospital Procedures and Outcomes

#### Crude Rates

The patients with and without DM underwent CA at similar rates (79.9% vs. 80.4%, *p* = 0.194), although the patients with DM had lower PCI rates (46.3% vs. 49.3%, *p* < 0.001). Among the patients with DM, there was a higher rate of CABG (20.2% vs. 18.2%, *p* < 0.001). (Table 2)

The crude rate of MACCE was lower in the patients with DM (40.8% vs. 43.1%, *p* < 0.001), while the in-hospital mortality rates were similar (35.8% vs. 36.4%, *p* = 0.215). The patients with DM also had lower crude rates of acute CVA (3.7% vs. 4.3%, *p* = 0.004) and cardiac complications such as coronary artery dissection (0.7% vs. 1.7%, *p* < 0.001) and pericardial effusion (2.5% vs. 3.1%, *p* < 0.001). The patients with DM were less likely, during hospitalization, to have VF (10.5% vs. 15.1%, *p* < 0.001) and VT (19.0% vs. 20.3%, *p* = 0.001).

However, the patients with DM had significantly longer hospital stays compared to those without DM, with a mean length of 10.4 days versus 9.6 days, respectively (*p* < 0.001). Additionally, the mean total hospital charge was higher for patients with DM at USD 252,202 compared to USD 239,538 for patients without DM (*p* < 0.001) (Table 1).

**Figure 2:**
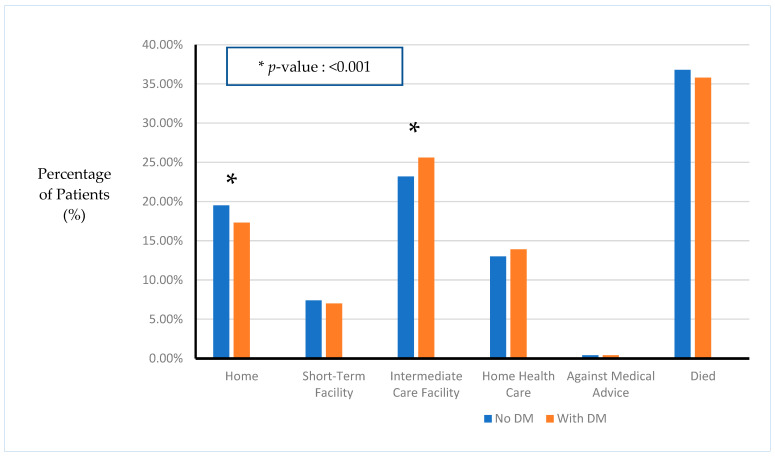
Disposition of patients based on diagnosis of diabetes.

Furthermore, the patients with DM had a slightly lower likelihood of being discharged home (17.3% vs. 19.5% for non-DM patient, *p* < 0.001) and a higher likelihood of being discharged to an intermediate care facility (25.6% vs. 23.2% for non-DM patients, *p* < 0.001) (Figure 2).

### 3.2. Adjusted Analysis

Following adjustment for baseline demographics and comorbidities, we did not observe a significant difference in the invasive procedures performed on patients with DM with similar odds of CA(aOR) 0.96 (95% CI: 0.91–1.01, *p* = 0.136), PCI, aOR 1.01 (95% CI: 0.97–1.06, *p* = 0.567), or CABG, aOR 0.96 (95% CI: 0.91–1.02, *p* = 0.211) at similar rates compared to patients without DM. There were also no differences regarding the aOR for mechanical ventilation (aOR 1.01, 95% CI: 0.96–1.05, *p* = 0.790) or circulatory support (aOR 0.97, 95% CI: 0.93–1.02, *p* = 0.232).

**Figure 3:**
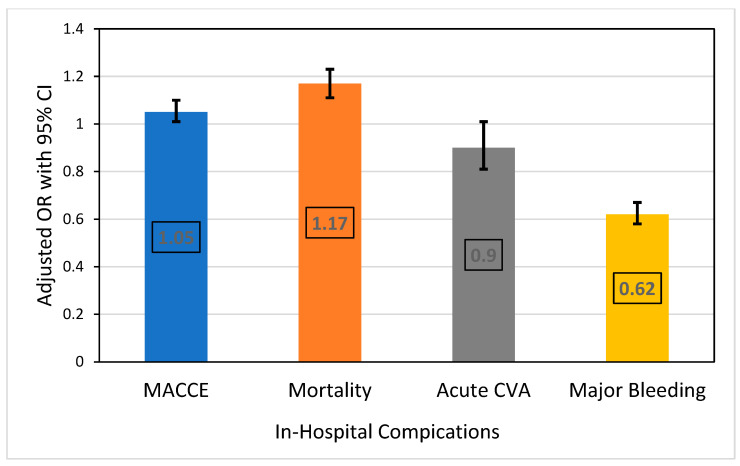
Adjusted odds ratio for in-hospital procedure complications in acute myocardial infarction patients with diabetes mellitus and polyvascular disease complicated by cardiogenic shock. Reference: no DM; adjusted for age, gender, hospital bed size, region and location/teaching status, STEMI, CABG, PCI, angiography, thrombolysis, mechanical ventilation, circulatory support, VF, VT, AF, HF, hypertension, valvular heart disease, smoking status, chronic liver disease, anemia, thrombocytopenia, coagulopathies, and malignancies.

In terms of in-hospital outcomes (Table 3 and Figure 3), the patients with DM had higher odds of MACCE, with an aOR of 1.05 (95% CI: 1.01–1.10, *p* = 0.020), and mortality with an aOR of 1.17 (95% CI: 1.11–1.23, *p* < 0.001). The odds of acute CVA did not significantly differ, with an aOR of 0.90 (95% CI: 0.81–1.01, *p* = 0.065). However, the patients with DM had significantly lower odds of major bleeding, with an aOR of 0.62 (95% CI: 0.58–0.67, *p* < 0.001).

## 4. Discussion

Our study involved 39,140 patients with AMI complicated by CS and known polyvascular disease, with the aim of assessing the differences in the management and outcomes of patients with and without DM. The findings revealed important insights into the complex interplay between diabetes, polyvascular disease, and acute cardiovascular events. The main findings of our study are as follows. First, the population of patients with DM was generally younger and had a higher proportion of females compared to that of the patients without DM. Second, the DM patients exhibited a higher burden of comorbidities, including heart failure, hypertension, and chronic kidney disease. Third, the patients with DM were less likely to present with STEMI, VF, and VT, but had similar rates of cardiac arrest, experienced longer hospital stays, and incurred higher total hospital charges. The patients with DM also had lower crude rates of PCI but higher rates of CABG, although the utilization of PCI and CABG was similar between the groups in the multivariable analyses. Finally, after adjustments, the patients with DM showed a worse prognosis, with a 17% increased mortality rate and higher risks of major adverse cardiovascular and cerebrovascular events.

The presence of polyvascular disease further complicates the prognosis for patients with DM. Polyvascular disease, defined as atherosclerosis in two or more major arterial beds, is an independent predictor of major adverse cardiovascular events and all-cause mortality in patients with AMI and DM [11,12]. Prior studies have shown that patients with DM and AMI, as well as those with DM and CS, face significantly worse outcomes, including higher mortality and increased rates of MACCE [13,14]. However, the role of DM in populations with a higher comorbidity burden, specifically those with polyvascular disease, remains undefined. Our results expand on these findings by focusing on a population with polyvascular disease, showing that DM significantly impacts outcomes in this high-risk group. Specifically, we found that the combination of DM and polyvascular disease leads to significantly worse outcomes, including increased mortality risk and higher rates of MACCE. 

Our findings of younger age and higher percentage of females in the DM group align with several previous studies [8,15,16]. A previous study encompassing 72,765 patients with myocardial infarction and associated cardiogenic shock reported that the population of patients with DM was on average 1.3 years younger than that of the patients without DM and had a higher proportion of females (38.1% vs. 35.5%) [8]. Similarly, in a study of elderly patients with myocardial-infarction-related cardiogenic shock, the population of patients with DM was found to have a higher proportion of females than that of the patients without DM (44.9% vs. 35.4%, *p* < 0.001) [15]. This was also noted in the SHOCK Trial Registry, where Shindler et al. noted a higher proportion of females among patients with DM with CS complicating AMI (49% vs. 36%, *p* < 0.001) [16]. This suggests that diabetes may accelerate the development of severe cardiovascular complications, and may lead to an earlier onset of AMI and CS, particularly in women.

Additionally, the patients with DM in our study exhibited a higher prevalence of comorbidities. This is consistent with previous findings, which showed significantly higher rates of heart failure, hypertension, chronic kidney disease, obesity, anemia, and chronic liver disease among patients with DM [8]. The high prevalence of comorbidities in patients with DM can be attributed to several factors. Chronic hyperglycemia, a hallmark of diabetes, contributes to the development of microvascular and macrovascular complications, increasing the risk of comorbidities such as heart failure, chronic kidney disease, and hypertension. Additionally, diabetes is often associated with other risk factors, like obesity and dyslipidemia, which further exacerbate the risk of developing comorbid conditions. The inflammatory and oxidative stress pathways that are involved in diabetes also contribute significantly to the development of these complications [2,17].

Furthermore, our study also highlighted significant differences in healthcare utilization and treatment patterns between patients with and without DM, particularly in the context of polyvascular disease. The patients with DM experienced longer hospital stays and incurred higher total hospital charges. This finding aligns with previous research on patients with AMI complicated by CS, albeit in a different population [8,17]. While our study specifically examined patients with polyvascular disease, both investigations consistently demonstrate the increased healthcare utilization and economic burden associated with DM in the context of AMI and CS. 

Interestingly, our study showed that the patients with DM were less likely to present with STEMI, VF, and VT, but that they demonstrated similar cardiac arrest rates. This finding is supported by previous studies, which reported that the percentage of STEMI was lower in patients with DM (40.8%) compared to patients without DM (49.4%) [18]. Similarly, Echouffo-Tcheugui et al. found that STEMI presentation occurred in 56.8% of patients with DM versus 65.4% of patients without DM [8]. Further supporting our findings, Mhaimeed et al. conducted a comprehensive analysis using the NIS database to assess the impact of diabetes on sudden cardiac arrest (SCA) in patients hospitalized for STEMI. Their study revealed that SCA significantly increased, from 4% in 2005 to 7.6% in 2018, in patients with DM, compared to an increase from 3% to 4.6% in patients without DM during the same period. Additionally, they found that diabetes was associated with an increased risk of SCA, with an adjusted odds ratio of 1.432 (95% CI: 1.336–1.707) [19].

Our findings align with previous research on revascularization patterns in patients with DM with AMI and CS, which observed lower rates of PCI but higher rates of CABG [8]. Additionally, Thoegersen et al. found that while there was no significant difference in the overall revascularization rates between patients with or without DM (85% vs. 87%, *p* = 0.454), patients with DM had higher rates of CABG (10.3% vs. 5.7%, *p* = 0.004) and lower rates of PCI (90.6% vs. 96.2%, *p* < 0.001). This suggests that patients with DM have a higher prevalence of multivessel disease [20,21], which may explain the observed differences in revascularization patterns.

Lastly, our finding of higher adjusted risk of MACCE and mortality are consistent with several previous studies [8,9,22]. For instance, a previous analysis found that diabetes was associated with increased in-hospital mortality in patients with acute myocardial infarction complicated by cardiogenic shock (adjusted OR 1.18, 95% CI 1.09–1.28; *p* < 0.001) [7]. Furthermore, Gąsior et al. reported higher in-hospital mortality rates for patients with DM compared to patients without DM (61.4% vs. 55.9%, *p* = 0.001) [22]. Our results demonstrated similar adjusted mortality rates for patients with DM in patients with MI complicated by CS in patients with polyvascular disease. However, this was in the setting of higher absolute mortality rates for patients with DM in our cohort compared to prior cohorts of patients with MI and CS, irrespective of polyvascular disease. Specifically, our study found that the patients with DM had a 17% increased mortality risk (adjusted OR 1.17, 95% CI: 1.11–1.23, *p* < 0.001) and a slightly higher risk of major adverse cardiovascular and cerebrovascular events (adjusted OR 1.05, 95% CI: 1.01–1.10, *p* = 0.020). This highlighted that DM remains prognostically significant, despite its higher baseline comorbidity burden, including many comorbidities that may be worsened by comorbid DM. 

However, there is an apparent discrepancy between the crude and adjusted outcomes for the patients with and without diabetes in our study. As shown in Table 2, we found that the patients with DM had a slightly lower crude rate of MACCE (40.8% vs. 43.1%, *p* < 0.001) than and similar in-hospital mortality (35.8% vs. 36. 4%, *p* = 0.215) to the patients without DM. However, after adjusting for baseline characteristics and comorbidities, DM was associated with higher odds of MACCE (aOR 1.05, 95% CI: 1.01–1.10, *p* = 0.020) and mortality (aOR 1.17, 95% CI: 1.11–1.23, *p* < 0.001). This seemingly paradoxical outcome can be explained by several factors. First, the patients with DM in our study were significantly younger (69.5 vs. 72.1 years, *p* < 0.001) and had a different comorbidity profile compared to the patients without DM. The younger ages of the DM patients may have contributed to their lower crude event rates. Second, the patients with DM had more comorbidities, such as heart failure, hypertension, and chronic kidney disease, which could have affected their outcomes. When these factors were accounted for in the multivariate analysis, the true impact of DM on outcomes became apparent.

Thus, our study has several practical implications for patient management. Patients with DM with AMI complicated by CS and polyvascular disease are at high risk, with longer lengths of hospital stay and higher inpatient costs, which should be closely monitored. The early identification of these patients can help focus care and resources on this vulnerable population. Further research is needed to develop and evaluate targeted interventions for this high-risk patient population, with the goal of improving both short-term outcomes during hospitalization and long-term cardiovascular prognosis.

Although this study offers important findings, several limitations were associated with the retrospective analyses of administrative databases, such as the NIS. The ICD-10 codes may have caused potential diagnostic or procedural coding errors or omissions. The NIS captured inpatient stays rather than individual patients, potentially resulting in duplicate counts for patients with multiple admissions during the study period. This could have led to an overestimation of certain events or outcomes. Moreover, the database provided limited clinical data on various types of information, including disease severity, specific treatment modalities, and long-term follow-up, to determine the relationship between or the effects of clinical variables on the outcome. The study also could not account for variations in hospital care quality or regional differences in healthcare delivery. Despite these limitations, the large sample size and nationally representative nature of the NIS database provided important population-level insights into the impact of diabetes and polyvascular disease on outcomes in patients with acute myocardial infarction complicated by cardiogenic shock.

## 5. Conclusions

In conclusion, diabetes mellitus significantly impacted outcomes in patients with AMI complicated by CS and polyvascular disease. In this study, we found that the patients with DM with this complex condition experienced longer hospital stays, incurred higher healthcare costs, and faced an increased risk of MACCE. Notably, the study highlighted a higher adjusted risk of in-hospital mortality for patients with DM, underscoring the critical need for targeted interventions and specialized care strategies for this high-risk population. These findings emphasize the importance of the early identification and aggressive management of patients with DM presenting with AMI and CS, particularly those with polyvascular involvement. 

## Figures and Tables

**Table 1:** Baseline demographic and clinical characteristics of acute myocardial infarction patients with polyvascular disease complicated by cardiogenic shock, stratified by diabetes status.

	No DM	With DM	*p*-Value
NIS discharge weight	18,000	21,140	<0.001
Mean age, years	72.1	69.5	<0.001
Female, %	34.2	36.7	<0.001
Ethnicity, %			<0.001
White	78.0	62.3	
Black	9.2	13.3	
Hispanic	5.9	13.8	
Asian	2.8	5.6	
Native	0.6	0.9	
Other	3.5	4.1	
Hospital region, %			<0.001
Northeast	15.4	13.8	
Midwest or North Central	23.8	22.5	
South	40.3	38.8	
West	20.5	24.9	
Hospital bed size, %			0.374
Small	13.6	13.3	
Medium	27.0	27.6	
Large	59.4	59.1	
Hospital location/teaching status, %			<0.001
Rural	4.2	3.5	
Urban non-teaching	17.4	19.1	
Teaching	787.4	77.4	
Median ZIP income			<0.001
1st Quartile	29.0	31.3	
2nd Quartile	26.9	27.0	
3rd Quartile	24.3	23.5	
4th Quartile	19.8	18.2	
Primary Expected Payer, %			<0.001
Medicare	71.3	72.6	
Medicaid	7.0	7.9	
Private Insurance	16.5	14.7	
Self-pay	2.8	2.7	
No charge	0.1	0.1	
Other	2.3	2.1	
Record Characteristics, %			
STEMI	50.0	38.2	<0.001
Cardiac arrest	8.7	9.0	0.238
Ventricular fibrillation	15.1	10.5	<0.001
Ventricular tachycardia	20.3	19.0	0.001
Length of stay, days, mean	9.6	10.4	<0.001
Total charge, USD, mean	$239,538	$252,202	<0.001
Comorbidities, %			
Heart failure	67.8	75.3	<0.001
Valvular heart disease	25.4	23.1	<0.001
Hypertension	82.3	92.8	<0.001
Atrial fibrillation	37.5	34.7	<0.001
Smoking	49.0	37.5	<0.001
Dementia	6.2	5.7	0.040
Chronic kidney disease	57.8	78.2	<0.001
Obesity	10.8	22.1	<0.001
Anemia	49.2	55.6	<0.001
Thrombocytopenia	16.9	16.4	0.164
Coagulopathy	6.9	5.9	<0.001
Chronic liver disease	0.9	1.2	<0.001
Hematologic malignancy	1.8	0.9	<0.001
Solid malignancy	2.9	1.8	<0.001
Metastatic malignancy	1.3	0.5	<0.001

**Table 2:** In-hospital procedures, complications, and outcomes in acute myocardial infarction patients with polyvascular disease complicated by cardiogenic shock, stratified by diabetes status.

	No DM	With DM	*p*-Value
NIS discharge weight	18,000	21,140	<0.001
In-hospital procedures			
Coronary angiography, %	80.4	79.9	0.194
PCI, %	49.3	46.3	<0.001
CABG, %	18.2	20.2	<0.001
Thrombolysis, %	0.3	0.3	0.651
Mechanical ventilation, %	46.8	46.4	0.456
Circulatory support (inc. IABP, LV assist device and ECMO), %	43.1	42.6	0.382
In-hospital complications			
MACCE, %	43.1	40.8	<0.001
Mortality, %	36.4	35.8	0.215
Acute CVA, %	4.3	3.7	0.004
Cardiac complications			
Coronary artery dissection, %	1.7	0.7	<0.001
Pericardial effusion (including tamponade), %	3.1	2.5	<0.001
Tamponade, %	1.1	0.8	0.004
Dressler’s syndrome, %	0.2	0.2	0.373
Post-MI angina, %	0.5	0.5	0.548
Intracardiac thrombus, %	0.6	0.3	<0.001
Mechanical complications, %	1.0	0.2	<0.001
Vascular complications, %	0.7	0.3	<0.001
Major GI bleeding, %	11.7	7.7	<0.001
GI bleeding, %	7.7	5.9	<0.001
Procedural related bleeding, %	3.2	1.5	<0.001
Intracerebral hemorrhage, %	0.6	0.5	0.127
Retroperitoneal bleeding, %	0.7	0.2	<0.001
Post-procedural shock, %	0.4	0.3	0.024

**Table 3:** Multivariate analysis showing adjusted odds ratios for in-hospital procedures and complications in acute myocardial infarction patients with diabetes mellitus and polyvascular disease complicated by cardiogenic shock.

Outcome	aOR (95% CI)	*p* Value
In-hospital procedures		
Coronary angiography	0.96 (0.91–1.01)	0.136
PCI	1.01 (0.97–1.06)	0.567
CABG	0.96 (0.91–1.02)	0.211
Thrombolysis	0.97 (0.67–1.40)	0.878
Mechanical ventilation	1.01 (0.96–1.05)	0.790
Circulatory support (inc. IABP, LV assist device and ECMO)	0.97 (0.93–1.02)	0.232
In-hospital complications		
MACCE	1.05 (1.01–1.10)	0.020
Mortality	1.17 (1.11–1.23)	<0.001
Acute CVA	0.90 (0.81–1.01)	0.065
Major bleeding	0.62 (0.58–0.67)	<0.001

Reference: no DM; adjusted for age, gender, hospital bed size, region and location/teaching status, STEMI, CABG, PCI, angiography, thrombolysis, mechanical ventilation, circulatory support, VF, VT, AF, HF, hypertension, valvular heart disease, smoking status, chronic liver disease, anemia, thrombocytopenia, coagulopathies, and malignancies.

## Data Availability

The data presented in this study are available on request from the corresponding author. The data are not publicly available due to privacy or ethical restrictions.

## Supplementary Materials

The following supporting information can be downloaded at: https://www.mdpi.com/article/10.3390/biomedicines12081900/s1. Table S1: ICD-10 codes for patient characteristics, in-hospital procedures and post-procedural complications.

## References

[1] Grundy S.M., Benjamin I.J., Burke G.L., Chait A., Eckel R.H., Howard B.V., Mitch W., Smith S.C., Sowers J.R. (1999). Diabetes and cardiovascular disease: A statement for healthcare professionals from the American Heart Association. Circulation.

[2] Leon B.M., Maddox T.M. (2015). Diabetes and cardiovascular disease: Epidemiology, biological mechanisms, treatment recommendations and future research. World J. Diabetes.

[3] Samsky M.D., Mentz R.J., Stebbins A., Lokhnygina Y., Aday A.W., Pagidipati N.J., Jones W.S., Katona B.G., Patel M.R., Holman R.R. (2021). Polyvascular disease and increased risk of cardiovascular events in patients with type 2 diabetes: Insights from the EXSCEL trial. Atherosclerosis.

[4] Gutierrez J.A., Scirica B.M., Bonaca M.P., Steg P.G., Mosenzon O., Hirshberg B., Im K., Raz I., Braunwald E., Bhatt D.L. (2019). Prevalence and Outcomes of Polyvascular (Coronary, Peripheral, or Cerebrovascular) Disease in Patients With Diabetes Mellitus (From the SAVOR-TIMI 53 Trial). Am. J. Cardiol..

[5] Kobo O., Cavender M.A., Jensen T.J., Kuhlman A.B., Rasmussen S., Verma S. (2023). Once-weekly semaglutide reduces the risk of cardiovascular events in people with type 2 diabetes and polyvascular disease: A post hoc analysis. Diabetes Obes. Metab..

[6] Kobo O., Contractor T., Mohamed M.O., Parwani P., Paul T.K., Ghosh R.K., Alraes M.C., Patel B., Osman M., Ludwig J. (2021). Impact of pre-existent vascular and poly-vascular disease on acute myocardial infarction management and outcomes: An analysis of 2 million patients from the National Inpatient Sample. Int. J. Cardiol..

[7] Samsky M.D., Morrow D.A., Proudfoot A.G., Hochman J.S., Thiele H., Rao S.V. (2021). Cardiogenic Shock After Acute Myocardial Infarction: A Review. JAMA.

[8] Echouffo-Tcheugui J.B., Kolte D., Khera S., Aronow H.D., Abbott J.D., Bhatt D.L., Fonarow G.C. (2018). Diabetes Mellitus and Cardiogenic Shock Complicating Acute Myocardial Infarction. Am. J. Med..

[9] Lindholm M.G., Boesgaard S., Torp-Pedersen C., Køber L., TRACE registry study group (2005). Diabetes mellitus and cardiogenic shock in acute myocardial infarction. Eur. J. Heart Fail..

[10] HCUP, National Inpatient Sample (NIS) (2012). Healthcare Cost and Utilization Project (HCUP).

[11] Verma S., Mazer C.D., Inzucchi S.E., Wanner C., Ofstad A.P., Johansen O.E., Zwiener I., George J.T., Butler J., Zinman B. (2021). Impact of polyvascular disease with and without co-existent kidney dysfunction on cardiovascular outcomes in diabetes: A post hoc analysis of EMPA-REG OUTCOME. Diabetes Obes. Metab..

[12] Bonaca M.P., Gutierrez J.A., Cannon C., Giugliano R., Blazing M., Park J.G. (2018). Polyvascular disease, type 2 diabetes, and long-term vascular risk: A secondary analysis of the IMPROVE-IT trial. Lancet Diabetes Endocrinol..

[13] Luo C., Chen F., Liu L., Ge Z., Feng C., Chen Y. (2022). Impact of diabetes on outcomes of cardiogenic shock: A systematic review and meta-analysis. Diab Vasc. Dis. Res..

[14] Jain V., Qamar A., Matsushita K., Vaduganathan M., Ashley K.E., Khan M.S., Bhatt D.L., Arora S., Caughey M.C. (2023). Impact of Diabetes on Outcomes in Patients Hospitalized With Acute Myocardial Infarction: Insights From the Atherosclerosis Risk in Communities Study Community Surveillance. J. Am. Heart Assoc..

[15] Gual M., Albert-Solé A., Maárquez M.G., Fernández C., Bernal J.L., Formiga F., Barrionuevo M.I., Sánchez-Salado J.C., Lorente V., Pascual J. (2020). Diabetes mellitus, revascularization and outcomes in elderly patients with myocardial infarction-related cardiogenic shock. J. Geriatr. Cardiol..

[16] Shindler D.M., Palmeri S.T., Antonelli T.A., Sleeper L.A., Boland J., Cocke T.P., Hochman J.S. (2000). Diabetes mellitus in cardiogenic shock complicating acute myocardial infarction: A report from the SHOCK Trial Registry. Should we emergently revascularize Occluded Coronaries for cardiogenic shock?. J. Am. Coll. Cardiol..

[17] Piccolo R., Franzone A., Koskinas K.C., Räber L., Pilgrim T., Valgimigli M., Stortecky S., Rat-Wirtzler J., Silber S., Serruys P.W. (2016). Effect of Diabetes Mellitus on Frequency of Adverse Events in Patients With Acute Coronary Syndromes Undergoing Percutaneous Coronary Intervention. Am. J. Cardiol..

[18] Dauriz M., Morici N., Gonzini L., Lucci D., Di Chiara A., Boccanelli A., Olivari Z., Casella G., De Luca L., Temporelli P. (2020). Fifteen-Year Trends of Cardiogenic Shock and Mortality in Patients with Diabetes and Acute Coronary Syndromes. Am. J. Med..

[19] Mhaimeed O., Pillai K., Dargham S., Al Suwaidi J., Jneid H., Abi Khalil C. (2023). Type 2 diabetes and in-hospital sudden cardiac arrest in ST-elevation myocardial infarction in the US. Front. Cardiovasc. Med..

[20] Kesani M., Aronow W.S., Weiss M.B. (2003). Prevalence of Multivessel Coronary Artery Disease in Patients With Diabetes Mellitus Plus Hypothyroidism, in Patients With Diabetes Mellitus Without Hypothyroidism, and in Patients With No Diabetes Mellitus or Hypothyroidism. J. Gerontol. Ser. A Biol. Sci. Med. Sci..

[21] Li L., Li G., Chen H., Feng Z., Zhang L., Chen L., Fan L. (2021). Role of Diabetes Mellitus in Acute Coronary Syndrome Patients with Heart Failure and Midrange Ejection Fraction Who Have Undergone Percutaneous Coronary Intervention: A 3-Year Case-Series Follow-Up Retrospective Study. DMSO.

[22] Gąsior M., Pres D., Gierlotka M., Hawranek M., Słonka G., Lekston A., Buszman P., Kalarus Z., Zembala M., Poloński L. (2012). The influence of diabetes on in-hospital and long-term mortality in patients with myocardial infarction complicated by cardiogenic shock: Results from the PL-ACS registry. Kardiol. Pol..

